# Molecular analysis of scats revealed diet and prey choice of grey wolves and Eurasian lynx in the contact zone between the Dinaric Mountains and the Alps

**DOI:** 10.1186/s12983-024-00530-6

**Published:** 2024-03-19

**Authors:** Elena Buzan, Hubert Potočnik, Boštjan Pokorny, Sandra Potušek, Laura Iacolina, Urška Gerič, Felicita Urzi, Ivan Kos

**Affiliations:** 1 Faculty of Mathematics, Natural Sciences and Information Technologies, University of Primorska, Glagoljaška 8, 6000 Koper, Slovenia; 2 Faculty of Environmental Protection, Trg mladosti 7, 3320 Velenje, Slovenia; 3 Biotechnical Faculty, University of Ljubljana, Jamnikarjeva 101, 1000 Ljubljana, Slovenia; 4 Slovenian Forestry Institute, Večna pot 2, 1000 Ljubljana, Slovenia; 5 Department of Chemistry and Bioscience, Aalborg University, Frederik Bajers Vej 7H, 9220 Aalborg, Denmark

**Keywords:** Dietary analysis, Non-invasive samples, Scats, Metabarcoding, *Canis lupus*, *Lynx lynx*

## Abstract

A comprehensive understanding of the dietary habits of carnivores is essential to get ecological insights into their role in the ecosystem, potential competition with other carnivorous species, and their effect on prey populations. Genetic analysis of non-invasive samples, such as scats, can supplement behavioural or microscopic diet investigations. The objective of this study was to employ DNA metabarcoding to accurately determine the prey species in grey wolf (*Canis lupus*) and Eurasian lynx (*Lynx lynx*) scat samples collected in the Julian Alps and the Dinaric Mountains, Slovenia. The primary prey of wolves were red deer (*Cervus elaphus*) (detected in 96% scat samples), European roe deer (*Capreolus capreolus*) (68%), and wild boar (*Sus scrofa*) (45%). A smaller portion of their diet consisted of mesocarnivores, small mammals, and domestic animals. In contrast, the lynx diet mostly consisted of European roe deer (82%) and red deer (64%). However, small mammals and domestic animals were also present in lynx diet, albeit to a lesser extent. Our findings indicate that the dietary habits of wolves and lynx are influenced by geographical location. Snapshot dietary analyses using metabarcoding are valuable for comprehending the behaviour and ecology of predators, and for devising conservation measures aimed at sustainable management of both their natural habitats and prey populations. However, to gain a more detailed understanding of wolf and lynx dietary habits and ecological impact, it would be essential to conduct long-term genetic monitoring of their diet.

**Supplementary Information:**

The online version contains supplementary material available at 10.1186/s12983-024-00530-6.

## Introduction

Accurate inferences about the diets of wild predators are essential for understanding their ecosystem impacts and roles [1, 2] as well as for resolving conflicts between predators and humans. Apart from causing direct conflicts, such as predation on livestock, the presence of predators has significant impacts on wild prey species, for example ungulates, and must be considered in their management [3, 4]. Primarily, predation, particularly by carnivores, is an important cause of ungulate mortality, which additionally impacts their abundance and density as well as population trends and demographic characteristics [5, 6]. The consequences or effects of predation may extend beyond prey abundance, potentially influencing demographic structure, physical condition, parasite load, genetic traits and behaviour of prey species [7–11]. The presence of predators makes prey more alert and alters their circadian activities, space use, group size, etc. [8, 12–15].

The grey wolf (*Canis lupus*), along with the Eurasian lynx (*Lynx lynx*), is the most important predator of ungulates in temperate forests [5, 16, 17]. The grey wolf (hereafter referred to as “wolf”) is considered an opportunistic predator [18, 19], that generally consumes ungulate species, but may change its diet depending on the availability of prey and feed on the most abundant species [20–23]. Previous studies in North America and central Europe have shown that wolves rely predominantly on large wild ungulates and some other medium-sized wild mammals [18, 24–28]. This is in contrast to southern Europe, where in lack of large wild ungulates, they mainly prey on medium-sized wild ungulates and domestic animals (livestock) [25, 29–35].

Eurasian lynx (hereafter referred to as “lynx”) is a highly specialised ambush predator that follows an opportunistic foraging strategy similar as the wolf. It hunts multiple prey species, but often selectively chooses smaller ungulates [36]. Its main prey species is European roe deer (*Capreolus capreolus*), which can account for up to 75% of its diet [37–44]. However, despite their strong ties to ungulates, lynx and wolves have a wide range of alternative prey, including beavers (*Castor* sp.), small mammals, mustelids and birds that can supplement ungulates when they are scarce [17, 27, 45–48].

In Slovenia, studies on wolf scats revealed that their main prey is red deer (*Cervus elaphus*), which accounts for 85% of the biomass consumed [49–51]. In the Dinaric Mts, the mortality rate of red deer due to wolf predation was found to be 7.8% [50], and the wolf has been recognised as an important selector (i.e., preying mainly on young animals), thus influencing the sex and age structure of the red deer population [49, 50, 52]. In the same region, cervids account for 50–99% of the prey for lynx. However, in areas where both European roe deer and red deer occur, lynx preferentially prey on European roe deer, which can account for up to 80% of the total biomass consumed. The average frequency at which a single lynx preys on European roe deer is 47.8 individuals/year, i.e., one roe deer every 7.6 days or 0.2 roe deer/100 ha/year, which is about 8% of the local roe deer population [53, 54].

Scats can provide a snapshot of the predator diet in a non-invasive manner and are easier to collect compared to other types of biological samples [55]. Traditionally, scat-based dietary analysis has relied on mechanical processing and sorting of scat remains [56–60]. Diet analysis by mechanical sorting is based on macro- or microscopic morphological identification of food remains and taxonomic identification of undigested food debris in scats (or stomachs). However, if the particles are too small (e.g., bone fragments) or very similar to each other (e.g., hairs belonging to related/similar species), morphological methods are ineffective [61–64], and are subjected to systematic biases [59, 65, 66]; in particular, rare species or species lacking indigestible hard parts are often overlooked or misidentified [57]. DNA-based methods for detecting prey in predators' scats are therefore a valuable alternative to conventional microscopic approach [67]. Indeed, in comparison to traditional morphological/microscopic techniques, DNA analyses of scats have become increasingly reliable due to significantly higher prey detection rate [55, 61, 68, 69], lower observer bias [68], and higher taxonomic resolution with more reliable separation of closely related taxa [68, 70, 71].

Determination of degraded DNA in scats by conventional molecular techniques based on Sanger chemistry [72] is very difficult because such analysis requires very well-preserved DNA. In scat samples, a number of factors, including the environment, the age of scats, and various degradation processes, can importantly affect the quantity and quality of DNA [73]. In addition, isolated prey DNA may be present in fewer replicates (copies), compared to the predator’s DNA, due to fragmentation and degradation by biochemical digestion processes and is usually contaminated with genetic material from predators. Therefore, modern next-generation sequencing methods represent a better alternative and enable rapid and reliable taxonomic identification of prey by DNA barcoding, even when DNA is present in a low copy number and is poorly conserved and heavily fragmented [74–78]. The barcoding system is based on the use of short DNA fragments informative enough to accurately identify the species. Mitochondrial DNA (mtDNA) is the predominant DNA region used in taxonomic and phylogenetic studies to distinguish species [79]. Mitochondrial gene sequences exhibit low intraspecific variability compared to nuclear DNA but in most cases provide sufficient interspecific variation for taxonomic identification [80].

To identify prey species in the scats of predators, species-specific short fragments of mtDNA are amplified with primers specific to a broader prey taxon [68]. While DNA barcoding involves sequencing of one well-curated individual at a time, metabarcoding involves massive parallel sequencing of complex environmental samples (eDNA). Therefore, faecal DNA metabarcoding has become a commonly used method for diet analysis [76–78, 81–85]. It usually involves workflow extraction of the total DNA from scat samples, DNA amplification with universal primers [86], and next-generation sequencing of the amplified products. This method saves time and allows accurate identification of amplified DNA sequences [87]. A unique feature of this approach, which complements and refines valuable traditional microscopic analyses of diets [87, 88], is the identification of species that are rarely or only seasonally represented in diets or may be overlooked by traditional methods [77, 89]. In addition, this methodology allows rapid and reliable taxonomic identification, even when DNA is fragmented, as even short DNA fragments can be fully sequenced [76–78, 81, 82, 84, 85].

In this study, we used DNA metabarcoding to provide a snapshot dietary profile for wolves and lynx using their scats, opportunistically collected in the Julian Alps and the Dinaric Mts (Slovenia, Central Europe). We compared dietary habits between species and between regions. Moreover, we did the comparison between fresh (i.e., estimated to be less than two days old) and partially degraded scats, aiming to ensure that the obtained prey reads were suitable for downstream statistical analysis and to confirm that no biases were introduced due to the freshness of the scats analysed.

## Materials and methods

### Study site and sample collection

**Fig. 1:**
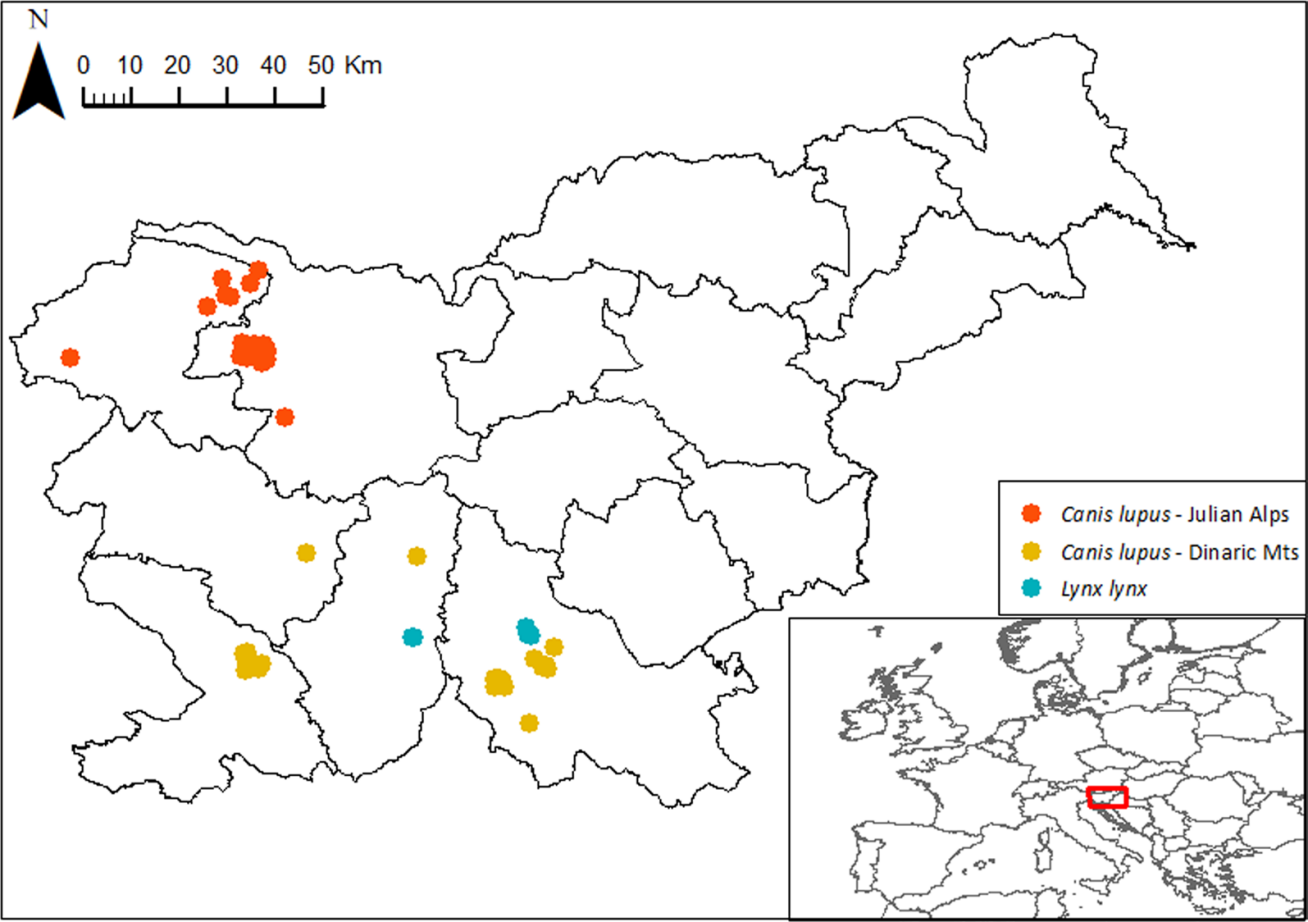
Geographic origin of wolf and lynx scats, collected in the Julian Alps and the Dinaric Mts (Slovenia), in the period 2019–2022 (for more information, see Additional file 1: Table S1)

In the study, we included 100 scat samples collected from spring to autumn 2019–2022 (88 wolf scats collected between 2020 and 2022, and 12 lynx scats collected between 2019 and 2022, respectively). Wolf scat samples were from two areas: the Julian Alps (*n* = 59) and the Dinaric Mts (*n* = 29). All lynx scat samples were from the Dinaric Mts (Fig. 1). Samples were collected by hunters or wildlife researchers and frozen in 70% ethanol solution at − 80 °C immediately after collection. Before analyses, all scats were taxonomically confirmed to be either wolf or lynx scats by large carnivore experts and genotyped using a set of species-specific recommended markers [89, 90] as part of the ongoing national wolf and lynx monitoring.

### DNA extraction and quality control

The QIAamp Fast DNA Stool Mini Kit (Qiagen, Germany) was used for DNA isolation following the manufacturer’s instructions. The kit is designed for the purification of DNA from scats and allows the isolation of total DNA from fresh or frozen samples. We repeated DNA isolation from the scat in 3 replicates using 220 ± 5 mg of sample. In parallel, a negative isolation control to which no faecal matter was added was also included. The concentration and purity of DNA obtained were measured with a 3.0 Qubit Fluorimeter using Invitrogen™—Qubit™ dsDNA BR Assay Kit (Life Technologies, Carlsbad, CA, USA).

### Library preparation and sequencing

The metabarcoding procedure involved the analysis of mtDNA 12S region with an expected amplicon length between 100 and 150 bp. The fragment was amplified using the Platinum Direct PCR Universal Master Mix Kit (ThermoFisher Scientific, USA), which contains a ready-to-use reaction mixture of Invitrogen Platinum II Taq Hot-Start DNA Polymerase, dNTPs, and green dye. We used primers V5-12S-F (5′-TTAGATACCCCACTATGC-3′) and V5-12S-R (5′-TAGAACAGGCTCCTCTAG-3′) [91, 92] at a concentration of 10 mM. All polymerase chain reactions (PCR) were performed in a total volume of 50 μl (details of PCR protocol are given in Additional file 1: Table S2). Negative PCR control using DNA-free water instead of template were included for each PCR triplicates. The amplicons from the triplicates were pooled and purified with magnetic particles Agencourt® AmPure® (Agencourt Bioscience Corporation, A Beckman Coulter Company, Beverly, MA, USA), following the manufacturer's instructions. Concentrations of pooled and cleaned amplicons were quantified by Qubit 3.0 fluorometry using Invitrogen™—Qubit™ dsDNA BR Assay Kit reagents. Samples were normalized to 3 ng and combined into a final library, which was again purified with Agencourt® AmPure® magnetic particles. For the separation, sizing, and quantification of dsDNA final library amplicons we used Agilent DNA High Sensitivity Kit on a 2100 Bioanalyzer (Agilent, Santa Clara, CA, USA). Specific barcodes were bound to the prepared libraries for individual identification using the Ion Xpress™ Plus Fragment Library Kit (ThermoFischer Scientific, USA), using a concentration of 100 ng amplified DNA/library to bind the barcodes. The barcoded libraries were normalised to a DNA mass of 5 ng and the individual libraries were pooled into a final library that was purified and prepared for sequencing according to the protocol described above. Library was multiplicated and banded with Ion Sphere particles (ISPs) using the Ion 520 & 530 Kit-OT2 reagent kit (ThermoFischer Scientific; cat. No.: A27751) according to the protocol for sequencing 400 bp long fragments on Ion Torrent One Touch 2 (OT2) and sequenced following the ThermoFischer Scientific platform instructions on Torrent S5, using Ion 530 chip (ThermoFisher Scientific, USA).

### Bioinformatic processing and statistical analyses

Raw sequence reads were analysed using a bioinformatics pipeline designed to trim and demultiplex the sequence reads. *Fastq* files for each barcoded sample were processed using *QIIME2* [93]. The primer sequence was removed using *Cutadapt* (*qiime cutadapt*) [94]. We uploaded the data (the obtained nucleotide sequences) into the *DADA2* programme (*qiime dada2 denoise-pyro*) [95]. The trimming was set to: *–p-trim-left 0 and –p-trunc-len 0*. For each individual sample, we thus obtained a set of all unique sequences of the 12S region of the mitochondrial genome. Taxonomic assignment was carried out in the pipeline with RESCRIPt pipeline (*qiime rescript get-ncbi-data*) [96] followed by filtering low quality sequences, evaluating the taxonomy (*qiime rescript evaluate-taxonomy –i-taxonomies amniota-12S-ref-tax-derep.qza –o-taxonomy-stats amniota-12S-ref-tax-keep-eval.qzv*). The remaining unique sequences after the cleaning and taxonomic verification steps were referred to as amplicon sequence variance (ASVs). We performed a series of filtering and quality control measures on amplicon sequence variances. Sequence filtering for metabarcoding requires balancing errors of commission (falsely including a species that was not present) and errors of omission (falsely removing a species that was present). To this end, we kept the sequences that: i) matched to the reference database with *E* value < 1 × 10^–30^ and a minimum percentage of identity of 0.98 (taxa that could not be assigned to a species were manually re-checked with BLAST and assigned to a genus or family based on the percentage match of related taxa), and ii) had at least 3 reads for each taxon observed within the pooled PCR replicates.

Considering the strict filtering applied, we confidently retained species, even if they were rare, occurred in low counts and only constituted a small proportion of the reads in the scat, as long as it was not observed in blanks. We removed sequences identified as *Canis* spp. and *Lynx* spp., and those detected in no-template controls (mainly human contamination). Samples with less than 1500 total reads were also discarded (six wolf and one lynx scats).

Finally, we compared taxonomic assignments with known fauna of Slovenia [97] to replace non-regional species identified with BLAST with closely related regional taxa. This was necessary for some rodents that assigned to related congeners or confamilias sometimes with 100% match.

All statistical analyses were performed in R 4.2.2 [98]. Based on the presence/absence of each prey taxa in each scat sample we calculated the frequency of occurrence (FOO) to describe the occurrence of prey in wolf and lynx diet. FOO was calculated to determine, based on the number of samples, which prey species were present and how often. FOO was calculated as the number of scats in which a prey species occurred divided by the total number of scats analysed per each species and area.

### Freshness of scats

Based on the perceived freshness of each scat, as determined by scat collectors during fieldwork, we pooled them into two categories, i.e. fresh and partially degraded scats. Scats that appeared moist, with shiny mucus on the surface and strong odour, were classified by collectors as “fresh”, meaning they were deposited within 48 h prior to collection. All other analysed scat samples were classified and referred as “partially degraded” scats. Most experts who collected scats checked their designated area 1–3 times per week, which means that the age of a considerable number of scats was also controlled by the check interval. This is especially relevant for wolf scats, which were mostly found along forest roads and crossings. A total of 93 scats were included in the analysis, 68 fresh and 25 partially degraded. Since fresh scats have higher genotyping success, someone might assume that fresh scats not older that 48 h are necessary for high-quality metabarcoding outcome. To test whether this assumption holds true in our case, we performed a *Mann–Whitney* test to determine whether fresh and partially degraded scats yielded different numbers of wolf sequence reads, prey sequence reads, or average quantity of DNA (ng/μL) in a sample.

### Variation between wolf and lynx diet

Analysis of differences among prey species was based on the presence/absence of each prey taxa in each scat sample (Additional file 1: Table S3). We also created a relative read abundance (RRA) matrix, i.e., relative species composition for comparison analysis (Additional file 1: Table S4). Read counts were transformed into RRA data using the vegan package with *decostand* function with *Hellinger* transformation [99], aiming to standardise the sample total abundances and to use transformed species scores, which provides a good linear relation of their Euclidean distances with used dissimilarities.

To test the effects of predator species and area on the diet composition, we conducted a permutational multivariate analysis of variance (PERMANOVA) using the *adonis2* function in *vegan* R, with *Jaccard* distance for the presence/absence matrix and with *Bray–Curtis* distance for the RRA matrix, both with 999 permutations. We constructed PERMANOVA models for groups (predator species and area) with diet composition as a response to determine the marginal effect of groups (by = “margin”). Dissimilarities in dietary composition between groups of scat samples were quantified using *Jaccard* distance for the presence/absence matrix and the *Bray–Curtis* distance for the RRA matrix, with 999 maximum iterations through the *metaMDS* function in *vegan* [100]. We used *Bray–Curtis* dissimilarity on occurrence data because it is a more appropriate metric weighting the abundances of shared species, and *Jaccard* distance on RRA because it it is appropriate for (unweighted) presence-absence data. To identify which prey items may be involved in driving distribution patterns between groups we used the *envfit* function in *vegan* R with 999 permutations. For visualization of patterns, we used the nonmetric multidimensional scaling (*NMDS*) plot [101, 102] with an acceptable stress level of < 0.2 [103]. Additionally, to identify which prey items drove the observed interspecific and spatial dietary differences for each pairwise comparison of groups, we used similarity percentage (*SIMPER*) tests implemented in *vegan* R package with 999 permutations (for the presence/absence matrix), where *SIMPER* shows the main prey species contribution at least to 70% of the differences between groups [103].

## Results

### Sequencing summary and reads assignment

The 93 samples out of 100 were successfully analysed (one wolf sample was not successfully amplified, and five wolf and one lynx samples were discarded due to low read; Additional file 1: Table S1). We obtained 8,642,237 raw sequence reads after demultiplexing; 99.1% of these reads were from sample PCR products and 0.9% were from negative controls. The read counts per sample ranged from 178 to 345,328 with a mean of 79,809 and a median of 57,357. Out of the 208 ASVs initially identified, 103 were eliminated as they did not match any taxon with a minimum of 98% identity. The remaining 105 ASVs were further analysed, with 20 of them being attributed to the predator (wolf or lynx) and the remaining 85 being identified as prey taxa. Notably, three ASVs had to be discarded as they were identified as *Homo sapiens*, which was likely due to contamination.

Overall, the scat samples contained 26 different diet items representing 24 species from 20 genera (Additional file 1: Table S5), and had on average 47,215 diet item DNA sequences per sample (SE = 2353). The number of diet items per scat ranged from one to eleven (mean = 3.6, SD = 0.7; Additional file 1: Table S6). Due to the low specificity of the primers, in two cases we used genus instead of species, i.e., in the case of *Ovis* (possibly *Ovis aries* or *O. gmelini musimon*) and *Felis* (*Felis catus* or *F. silvestris*) (Additional file 1: Table S7).

The overall average sequence coverage was 31,050 reads, of which 22,116 (71%) were predator reads and 8,639 (29%) were prey reads, respectively. For fresh scats, the average number of reads was 32,922 (69% predator, 31% prey), while for partially degraded scats it was 24,544 (81% predator, 19% prey). The number of predator reads was not significantly higher in fresh (Me = 8866) than in partially degraded scats (Me = 8470) (Mann–Whitney test: W = 796; P < 0.79). Similarly, there was no significant difference (W = 927.5; P < 0.15) between the number of prey sequence reads for fresh (Me = 2065) and partially degraded scats (Me = 727) (Additional file 1: Figure S1).

### Interspecific dietary differences

**Fig. 2:**
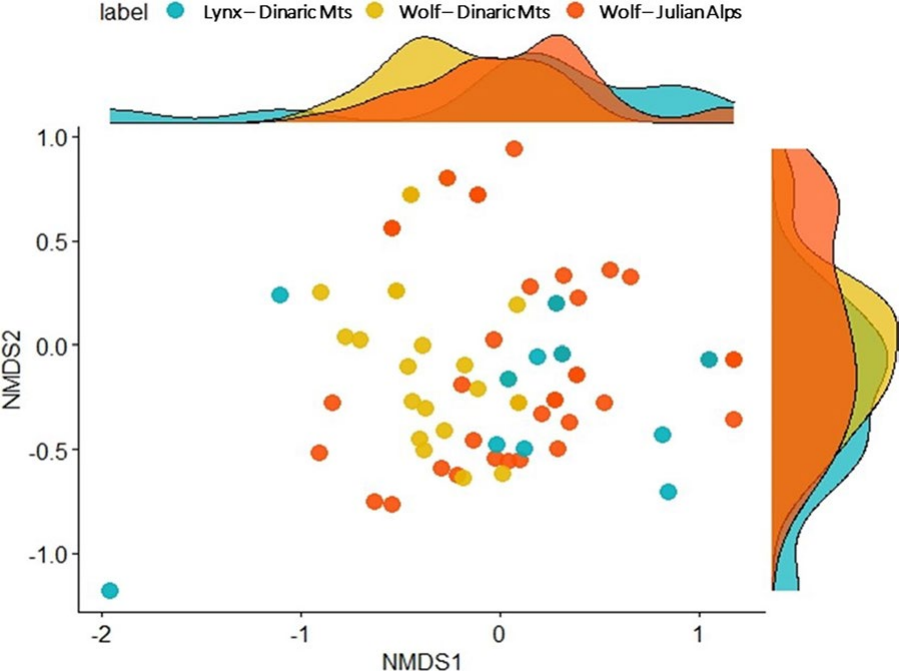
NMDS scatter histogram based on ‘prey item presence dataset’ of *Bray–Curtis* dissimilarity of wolf and lynx samples collected from different locations (*r*^2^ = 8.8%, *P* = 0.001), showing dietary distribution patterns

Our analysis indicated a significant relationship between the dietary patterns of wolf and lynx and the location where their scats were collected. Figure 2 shows the dietary distribution patterns found in wolf and lynx scats, based on their location and the presence or absence of different prey items.

### Wolf diet

**Fig. 3:**
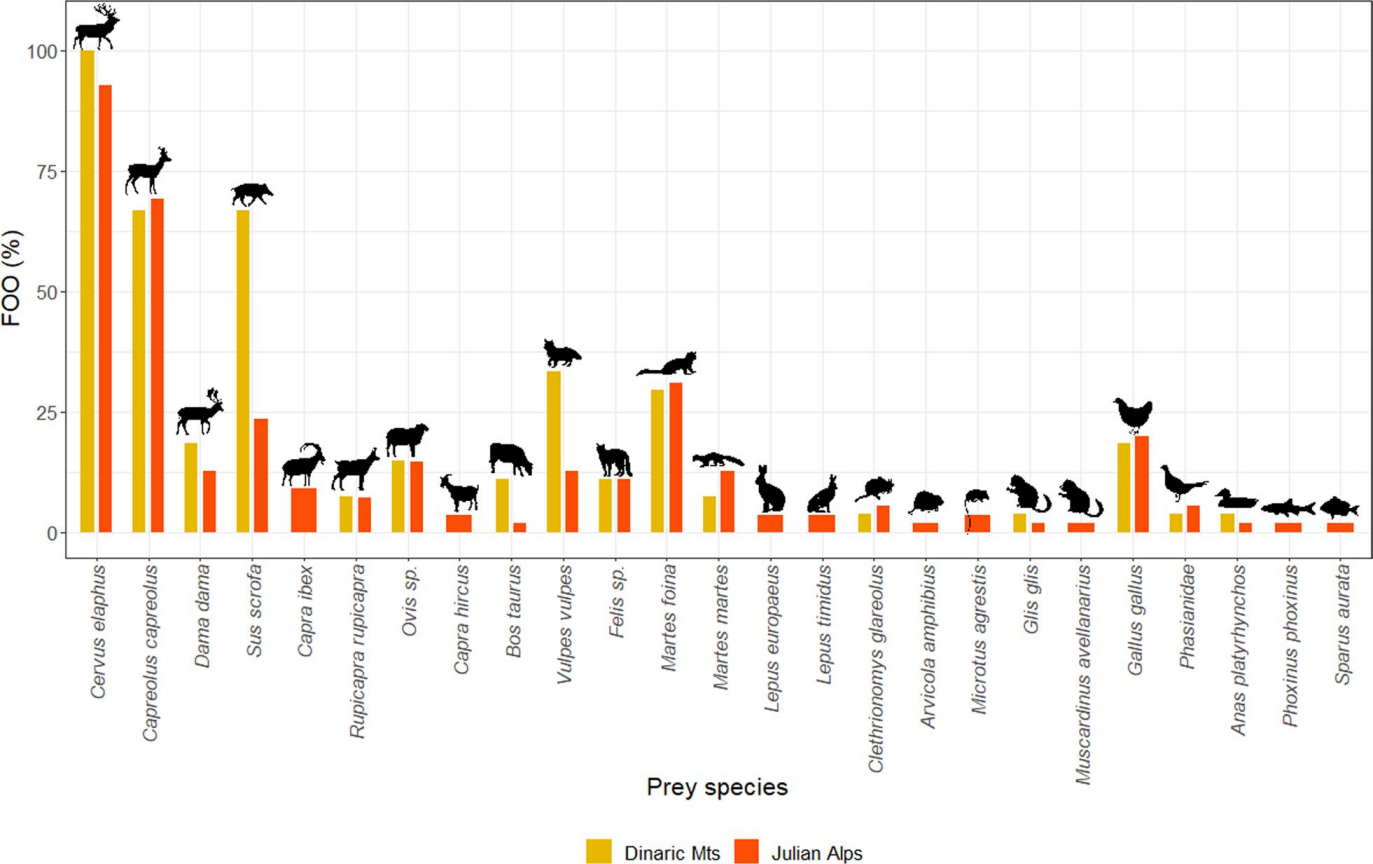
Frequency of occurrence (FOO) of prey species, whose DNA was present in the wolf scat samples examined, relative to the study area

In 82 sequenced wolf scat samples, we determined DNA of 25 potential prey species (Fig. 3; Additional file 1: Table S7). As expected, the highest FOO belongs to ungulates, especially red deer (the highest DNA occurrence in 51 samples (62%); red deer DNA was present in 78 samples, FOO = 93%), but also European roe deer (the highest occurrence in 16 samples (18%); present in 56 samples, FOO = 67%) and wild boar (*Sus scrofa)* (highest occurrence in 3 samples (3%); present in 31 samples, FOO = 37%). In wolf scats collected in Julian Alps, we found DNA of Northern chamois (*Rupicapra rupicapra*) in six samples and Alpine ibex (*Capra ibex*) in five samples, which is one of the few confirmations of predation on this species by wolves (i.e., also data from Gran Paradiso, Italy [104] and from undefined area in the Alps [2]). In this area (Jelovica), we identified DNA of mountain hare (*Lepus timidus*) in two scat samples. We also confirmed the presence of several small mammals’ DNA in individual wolf scat samples (Additional file 1: Table S7). The rodents whose DNA was detected in/on wolf’s scats were as follows: field vole (*Microtus agrestis*), bank vole (*Clethrionomys glareolus*), European water vole (*Arvicola amphibius*), hazel dormouse (*Muscardinus avellanarius*), and European edible dormouse (*Glis glis*).

We also detected several domestic animals in the diet of wolves: goats (*Capra hircus*), chickens (*Gallus gallus*), sheep, and cattle (*Bos taurus*) (Additional file 1: Table S7). However, their FOO were much lower compared to wild ungulates. Regarding the sheep, it cannot be conclusively ruled out that DNA identified does not originates from European mouflon. Indeed, the high degree of similarity in the nucleotide sequence of mtDNA within the analysed region of both *Ovis* species does not allow to confidently assign the sequence to either species, even more so, considering both species are present on the Julian Alps. Nonetheless, *Ovis* sp. DNA was detected with relatively low FOO (14.5%), suggesting that this species is not a primary food source for wolf in this area. Moreover, the presence of DNA of other domestic animals in the scats of wolves from the Julian Alps was confirmed only occasionally (cattle four times and poultry eighteen times). On the contrary, cattle were much more frequent in the diet of wolves in the Dinaric Mts (Vremščica), with FOO = 11%.

### Lynx diet

**Fig. 4:**
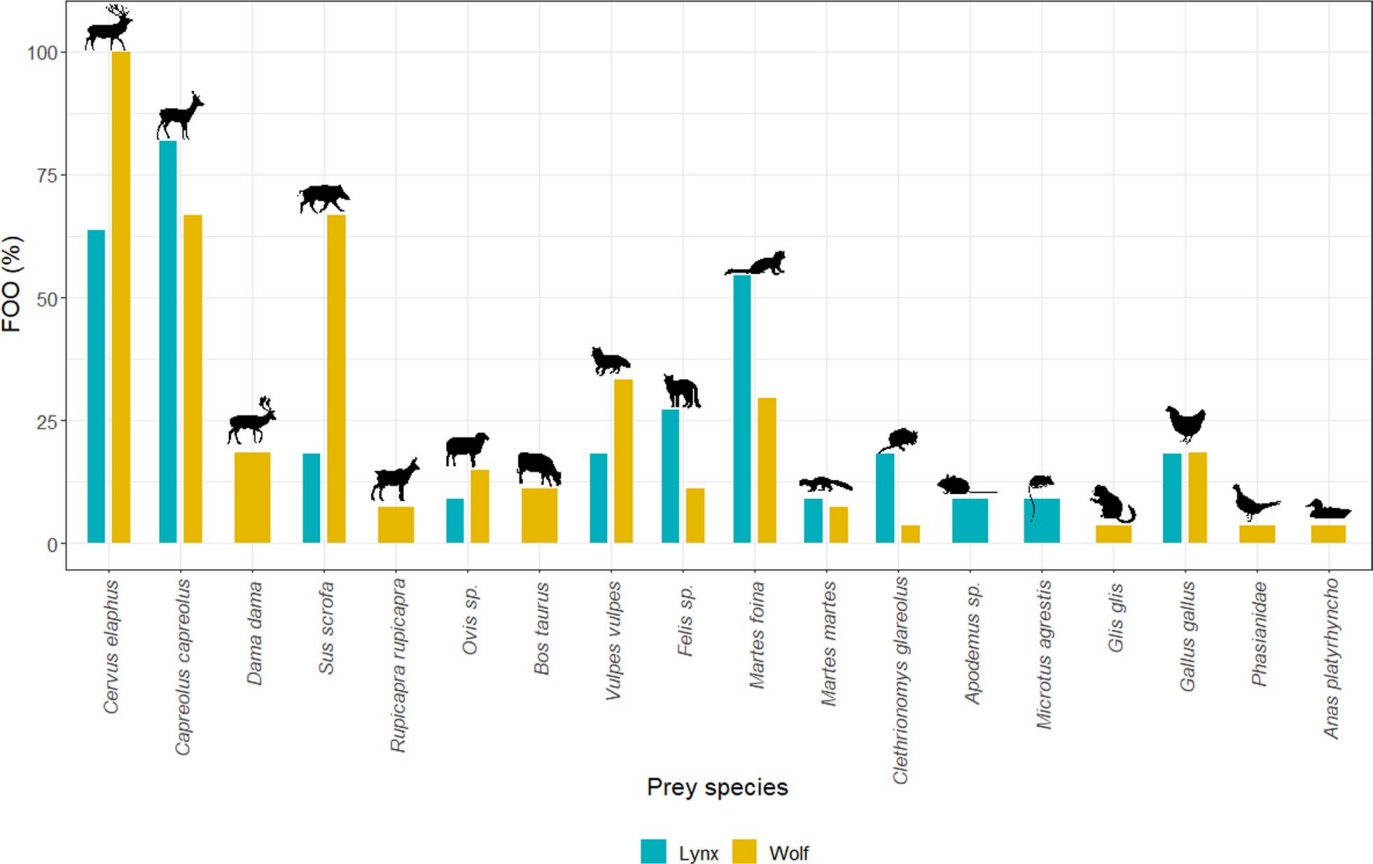
Frequency of occurrence (FOO) of prey species, whose DNA was present in the wolf and lynx scat samples examined in the Dinaric Mts

Considering the low abundance of lynx in Slovenia [105], we were only able to include 11 scats for this species. In this sample set, we determined DNA of 12 potential prey species (Fig. 4; Additional file 1: Table S7). Like for wolves, the most common prey species were ungulates, especially European roe deer (FOO = 82%). Red deer DNA was also detected in lynx scats (FOO = 64%). DNA of stone marten (*Martes foina*) was present in six samples (FOO = 54%), while that of pine marten (*Martes martes*) was present in one sample.

We also found the DNA of small mammals in individual samples of lynx scats: bank vole with FOO = 18% and field vole with FOO = 9% as well as a wood mouse species (*Apodemus* sp.) with FOO = 9%, concordantly with previous studies [54, 106]. We were able to detect high levels of red fox (FOO = 18%) and wild cat DNA (FOO = 27%) in lynx faeces, which, according to other studies, could also indicate predation of these mesocarnivores by the lynx [48]. However, it may also be due to their territorial behaviour, i.e., frequent marking on other animals' scats to mark territory.

We also detected domestic animals, i.e., chickens and sheep, in the diet of lynx (Fig. 4; Additional file 1: Table S7), although their FOO was relatively low (18% and 9%, respectively).

### Spatial variability in diet of both species

The PERMANOVA test for the effects of region/species (wolf Alps vs. Dinaric Mts; wolf and lynx from Dinaric Mts) on the dietary composition based on RRA showed significant differences of dietary variation among all groups (marginal *R*^*2*^ = 8.9%, *p* = 0.001); similar results were also obtained for the presence/absence dataset (marginal *R*^*2*^ = 5.6%, *p* = 0.002; Additional file 1: Table S8).

Considering differences in dietary composition among the three groups, the *envfit* test revealed statistically significant (*p* ≤ 0.05) contribution of 14 prey taxa in the presence/absence dataset (Additional file 1: Table S9, Additional file 2: Figure S2), and 11 prey taxa in the RRA dataset (Additional file 1: Table S9, Additional file 2: Figure S3).

### Spatial variation in wolf diet

The results clearly confirmed a difference in the presence of primary prey of wolves between the Julian Alps (Triglav National Park-TNP and Jelovica) and the Dinaric Mts (Vremščica, Velika gora and Mala gora) in terms of FOO (Fig. 3). *SIMPER* analyses (Additional file 1: Table S10) showed an average dissimilarity of 51.8% in prey selection between the two wolf groups. We found a cumulative contribution of the eight most influential species, with significant effects of wild boar (*p* = 0.01) and cattle (*p* = 0.01) (Additional file 1: Table S10, see Additional file 2: Figure S4 for ungulates abundance). The FOO of both species in the wolf diet was higher in the Dinaric Mts than in the Julian Alps (wild boar: 66.7% vs. 23.6%; cattle: 11.1% vs. 1.8%) (Additional file 1: Table S7). On the contrary, some species were found only in the diet of wolves from the Julian Alps: Alpine ibex (FOO = 9.0%), field vole (3.6%), mountain hare (3.6%), domestic goat (3.6%), hazel dormouse (1.8%), and water vole (1.8%) (Additional file 1: Table S7).

However, the dietary analysis also showed some similarity in the feeding preference of wolves in both areas, where red deer is the most important prey species (FOO of 92.8% in the Julian Alps and 100% in the Dinaric Mts), followed by roe deer (69.0% and 66.6%, respectively). In both areas, similar FOO were also found for martens, fallow deer (*Dama dama*), chicken, and sheep (Additional file 1: Table S7).

### Differences between wolf and lynx diet in the Dinaric area

We found a clear difference in the preference of primary prey of wolf and lynx in the Dinaric Mts (Fig. 4), where the average dissimilarity between the diet of both species was 57.8%. *SIMPER* analyses revealed seven prey species contributing to this difference (Additional file 1: Table S10), with a significant effect of wild boar (*p* = 0.02) and red deer (*p* = 0.014). Predation on wild boar (FOO = 66.7%) and red deer (100%) was higher by wolves than by lynx (18.2% in the case of wild boar and 63.6% for red deer). Frequency of other most influential prey species did not significantly differ between both carnivores, but we found DNA of European roe deer more frequently in lynx (81.8%) compared to wolves’ scats (66.7%); the same holds also for stone marten (54.5% vs. 29.6%), while FOO of chicken was comparable in both species (18.2% vs. 18.5%, respectively) (Additional file 1: Table S7).

Prey species detected only in the diet of wolves were fallow deer (18.5%), cattle (11.1%), and chamois (7.4%); besides, mallard (*Anas platyrhynchos*), undetermined bird species from the Phasianidae family, and edible dormouse were each found in only one wolf scat sample. On the other side, DNA of *Apodemus* sp. was detected in one lynx scat only (Additional file 1: Table S7).

The presence of domestic animals, i.e., DNA of chicken and *Ovis* sp. was found in wolf scats (18% and 15%, respectively), and in the lynx scats (18% and 9%, respectively), but not as a primary food source.

## Discussion

In general, our results confirmed that metabarcoding of scats is very useful tool for understanding the dietary characteristics of carnivores and the contribution of different food items in their diets. In our study, overall, wolves primarily preyed upon red deer (FOO = 94%), European roe deer (67%), and in the Dinaric Mts also upon wild boar (67%), while lynx preyed mainly on European roe deer (82%), mesocarnivores, and small mammals.

As all collected scats were rigorously verified by a carnivore experts and through genotyping to confirm their origin from either wolves or lynx, the possibility of incorrect identification of scats, i.e. the risk that reads attributed to wolves are misinterpreted and would in fact reflect predation on (feral) dogs, is rather minimal, as genetic differentiation between wolves and dogs has been routinely performed within national wolf monitoring protocols [107]. This is further supported by the fact that in Slovenia, there are almost no feral dogs, and the presence of free-ranging dogs in wolf habitats is very sporadic; moreover, there is almost no information on wolf predation on dogs. Our analysis also revealed the presence of red fox (*Vulpes vulpes*) and cat (*Felis* sp.*)* DNA in wolf and lynx scats. However, it is important to note that the presence of these reads does not necessarily indicate predation. This may be due to predator (in this case mesocarnivores) behaviour, such as territorial marking on the scats of other animals [108]. Nevertheless, it should also be considered that wolves can feed on mesocarnivores, and there have been cases of wolves attacking and killing cats that enter their territory [25]. Similarly, while not a common prey item for lynx, it is possible for them to feed on red foxes and domestic cats [25, 48, 109].

Our results on wolves are consistent with previous data obtained using traditional methods of microscopic determination of stomach and/or scat content in Slovenia [49] and Central Europe [27], where red deer are the main prey, but also in Southern Europe, where European roe deer and wild boar are found to be the main wild prey of wolves [27, 110]. However, our study also revealed notable dissimilarities in the dietary patterns of wolves in the studied areas of Dinaric Mts and Julian Alps, with wild boar being a major contributor to the observed spatial variability, which is related to differences in wild boar abundance in both studied areas as Julian Alps still has very low density of this species (Additional file 2: Figure S4). Although faecal metabarcoding is more sensitive in comparison with traditional microscopic analysis in determining qualitative composition of the diet (i.e., species presence), it cannot provide as precise insight into the qualitative aspect (i.e., frequency, volume share etc.). Moreover, the previous microscopic analysis of wolf diet in Slovenia did not focus on spatial differences, thus precluding us from definitively concluding whether observed differences between methods could be attributed to the varying detection capabilities of the methods employed. This leaves open the question of whether the distinctions we noted are due to of the inherent limitations or strengths of the analytical techniques used.

We also found that the frequency of occurrence of red deer DNA in the diet of wolves from the Dinaric Mts was significantly higher, reaching 100%. The observed variations in dietary composition between the study areas could be largely due to differences in prey densities/availability in these regions, i.e., due to higher population densities of both, red deer and wild boar in the Dinaric region [111], as prey availability is an important factor determining food selection patterns [81], or wolf/pack-specific preying behaviour. Predators generally select prey according to its availability and shift to consuming alternative food items when the primary food source is scarce [25, 112, 113]. As for instance, different factors (e.g. landscape, anthropogenic sources) could affect the wolf's feeding ecology, with the wolf using all available sources and showing flexibility in its attempts to survive [2].

The presence of mountain hare DNA in the scats from the Julian Alps could also result from misidentification with the more common and abundant brown hare (*Lepus europaeus*). This possibility should be indicated here, although mountain hares are present across Slovenian Alps [97]. The reason for this is that mtDNA region used in the metabarcoding analysis may not have enough variability to distinguish between the two species; moreover, introgression of mountain hare mtDNA into brown hare has already been reported in Europe [114, 115].

The presence of rodents’ DNA in wolf scats does not confirm directly that wolves had really fed on those species, because their DNA could appear incidentally on the surface of the scats (i.e., after urination or feeding). However, despite this cautionary note, the presence of DNA of three distinct small mammal species in two wolf scat samples from the Julian Alps implies that at least individual wolves prey on small mammals, which was previously also confirmed by microscopic scats analysis [47]. The detection of DNA of sea fish, gilthead bream (*Sparus aurata*), in the wolf faeces from the Julian Alps (Jelovica) is very interesting. Since secondary contamination of the sample can be excluded due to very rigorous preventive measures in the overall process of (pre)preparation of the samples (as described in the methods section), it is very likely that the wolf in this case fed on an anthropogenic food source (i.e., fish leftovers near summer houses or picnic areas). We also identified DNA of a common minnow (*Phoxinus phoxinus*), a small fish belonging to the carp family; however, since the species is of no interest for sport fishing and/or human consumption, we can neglect the possibility that the origin is the same as for bream, so stochastic or occasional feeding of wolves on this species (as well as on some other fish species living in streams) seems to be a plausible explanation.

As expected, our study confirmed a low frequency of occurrence of red deer in the diet of lynx, while European roe deer is a staple prey with the highest FOO. These findings align with previous knowledge demonstrating that lynx are not efficient predators of red deer (particularly not stags), due to their big size and risk for injuries related to their predation [16, 44]. However, lynx can effectively prey on red deer juveniles of both sexes, particularly during the calving season on neonates [116] as well as on weaker female yearlings and hinds [44, 45]. Lynx females typically give birth between April and June, which leads to elevated lactation requirements during springtime [117]. This could potentially result in increased predation pressure on prey species with higher ratio between nutritional value to predation effort, such as neonatal ungulates. Moreover, female roe deer in the late-gestation stage (April to early May) are also vulnerable targets for lynx [43, 118].

### Predation on domestic animals

A recovery tendency of the grey wolf population in the Dinaric and Alpine regions with its dispersal potential led to the spread of the species also into the human-dominated landscapes [119]. In this respect, we found relatively high FOO of chickens (18–20%) and, potentially, sheep (14.5–15%) in wolf scats in both areas, as well as cattle (11%) in the Dinaric Mts. A similar presence of livestock (10%) in the diet of wolves in the Dinaric Mts was also detected by previous traditional microscopic scat analysis [49]. Presumably, domestic animal depredation occurs especially in areas where livestock is abundant and unprotected. Although the reliance of wolves on wild ungulates as their main food source has been well documented [27], their low availability may lead wolves to increased motivation for preying upon other species, including domestic animals (livestock), especially in non-fenced areas and with inefficient husbandry techniques. Depredation of livestock can indeed occur frequently also in areas where natural prey is abundant, therefore management strategies to reduce livestock depredation should focus primarily on preventive measures. However, sustainable management of wild ungulates aimed at providing adequate population densities should also be one of the key issues in wolf conservation efforts [120], as it would not only sustain the population but potentially contribute to reduced human-wildlife conflicts.

There is evidence that lynx occasionally attack domestic animals [121]. However, previous studies have shown that cervids and edible dormice are the most frequently consumed prey items by lynx in the Dinaric Mts, while domestic animals make up only 16% of their diet, as indicated by traditional scat and stomach content analysis [54]. In our study, the frequency of occurrence of domestic animals in lynx diet was slightly lower (overall 9%), with chicken being the main domestic prey.

Knowledge on predators’ diet can provide important insight into the conflicts between carnivores and livestock breeders and facilitate implementation of relevant mitigation programs. The dietary profiles of both wolves and lynx suggest that they may occasionally prey on domestic animals such as young cattle (calf), sheep, goats, and chickens. Indeed, we found DNA of various domestic animals in wolf and lynx scats, which supports previous findings on these domestic species being part of the diet of both studied carnivores [27, 121]. The secondary contamination of our samples with livestock DNA was unlikely due to the absence of any such DNA in the negative controls, the exclusion of BSA in PCR reactions, and their detection in individual samples across independent analysis (i.e., Additional file 1: Table S5). However, it is important to acknowledge that the presence of domestic animal DNA in the scat samples of carnivores may also result from the consumption of carcasses and thus not necessarily confirms predation events.

### Metabarcoding of scats for diet analysis

DNA metabarcoding holds great potential as an emerging molecular technique, but caution is needed before drawing ecological conclusions [74, 122]. One of the key benefits of DNA metabarcoding compared to traditional sorting methods is its ability to provide high taxonomic resolution. Although we targeted vertebrates, we were not able to assign all reads at the species level. This limitation is likely due to the use of a single short marker, which is approximately 100 base pairs in length.

The degree of DNA degradation in scat samples limits the fragment length that can be successfully amplified to the range of 100–250 bp, which inevitably reduces taxonomic resolution [123]. However, our findings suggest that scats with assessed age of 3–5 days (indicated as partially degraded) can be relevantly used for metabarcoding. Indeed, there was no significant difference in the number of species detected and no considerable difference in the average DNA quantity between “fresh” and “partially degraded” group. Studies show that the number of days the scats are exposed to the natural environment has a negative effect on the detection of prey DNA, but the reported maximum degradation time varies between 5 and 60 days [83, 85]. Our results indicate that the collected scats were sufficiently preserved so that the detection of prey did not differ between scats up to 5 days old. Moreover, multigene approaches, such as the one described by Taberlet et al. [124], can additionally overcome limitations for species-specific identification and offer even better possibility of using degraded or partially degraded DNA.

DNA metabarcoding can provide more comprehensive results compared to microscopic analysis of scat samples. The comparison of the results from our study with previously published work done by microscopic determination of wolf scats in Slovenia [49, 51, 52] shows that metabarcoding is a much more sensitive approach. Indeed, 26 prey species were identified in our study, while mentioned microscopic analyses identified only the four main prey items in the wolf diet (red deer, European roe deer, wild boar, and domestic animals).

DNA metabarcoding, like most other diet analysis methods, cannot distinguish between active predation and scavenging, partial consumption of prey, and scats consumption (coprofagia) and/or their over-marking them by other carnivores. Thus, interpretation of the results in the context of the real spectrum of predated species and the resulting prey-predator interactions should be done with great caution and, whenever possible, in conjunction with other methods. For example, conventional methods such as field necropsies and examination of bite wounds can directly confirm predation [74] as well as the demographic category, health status, and physical fitness of prey, but are generally only applicable to large prey such as ungulates. Moreover, camera traps can occasionally capture predating events [125], and GPS collars and the use of collar-mounted activity sensors can provide information on animal movements, behaviour and activity, including predation [126]. Thus, combining DNA metabarcoding of scats with conventional, non-molecular diet analyses and direct field studies may be the most promising solution to ensure a comprehensive understanding of predation/feeding behaviour and ecological aspects of predator–prey interactions, although we are aware of the time and cost constraints of this multifaceted approach.

FOO is the most commonly used measure of diet composition [56]. However, the proportion of prey, the day the scats were collected, and also the meal size could influence the FOO estimate. The loss of information is primarily associated with older scats from smaller meals, especially when the prey is present in low concentrations in the predator's diet. The study by Thuo et al. [83] demonstrated a significant impact of the number of days since consumption on the proportion of prey DNA detection in cheetah (*Acinonyx jubatus*) scats. In this study, they also found a positive correlation between meal sizes and the probability of prey detection; with increased meal size, the probability of prey detection also increased. Early studies using traditional method on wolf diet in Slovenia converted FOOs of prey items to relative prey biomass and determined numbers of each prey species using regression equations derived from captive feeding trails [49]. While some studies have demonstrated the ability of metabarcoding to quantify the relative biomass of prey species [127], most studies advice caution for direct correlation between biomass and reads [128]. A recent meta-analysis suggests the possibility of a weak quantitative relationship between biomass and read counts, albeit with a significant degree of uncertainty [129].

So far, it would be impossible to do something similar when using DNA metabarcoding. Nevertheless, although recent developments in DNA metabarcoding of scats currently provide primarily qualitative (rather than quantitative) insight into the diets of carnivores, the higher taxonomic resolution of such data makes them very valuable for understanding their feeding patterns and ecological inferences including smaller and/or marginal prey species.

## Conclusion

Our findings suggest that modern molecular genetic methods can retrospectively determine feeding behaviour of target species, e.g. large carnivores, and can contribute to science-based approach in wildlife management [130]. The rapid development of molecular genetic methods and tools in recent years has increased their relevance and led to a significant drop in the price of genetic analyses, making them affordable for the end-users as well as for implementation in everyday monitoring/research practises in wildlife management and research [131, 132]. In our study, we clearly showed that snapshot molecular study of scat samples of grey wolves and Eurasian lynx can provide very valuable insight into the diet of these two apex predators. We confirmed previously published data that primary prey of wolves and lynx are the most abundant wild ungulate species in relevant area.

### Supplementary Information

**Additional file 1:** **Table S1.** Study site and sample collection. **Table S2**. Details of PCR protocol. **Table S3.** Occurrence of prey taxa in wolf and lynx scats. **Table S4.** Relative read abundance (RRA) matrix (the proportion of identified reads assigned to each prey taxon for comparison analysis). **Table S5.** Prey taxa identity classification. **Table S6.** Results of DNA analysis of prey species presence in grey wolf and Eurasian lynx scats. **Table S7.** Frequency of occurrence of the important prey items in wolf and lynx diet. **Table S8.** Results of PERMANOVA, testing the effects of predator species and spatial variation on the changes of diet composition. **Table S9. **Prey taxa most responsible for driving differences in dietary composition between groups. **Table S10. **Results of the similarity percentage (SIMPER) analysis on interspecific and spatial dietary difference for wolf and lynx diet.

**Additional file 2:** **Figure S1.** Boxplots depicting the total number of predator and prey DNA reads for scat samples binned by the perceived degradation of the scat. **Figure S2.** NMDS plots of presence matrix Bray-Curtis dissimilarity of wolf and lynx scat samples, collected from different locations showing the prey taxa involved in driving distribution patterns. **Figure S3.** NMDS plots of RRA-based Bray-Curtis dissimilarity of wolf and lynx scat samples, collected from different locations showing the prey taxa involved in driving distribution patterns.** Figure S4.** Sampling locations in relation to density gradients of three ungulate (main prey) species in Slovenia:** A** *Sus scrofa,*** B** *Capreolus capreolus*,** C** *Cervus elaphus*.

## Data Availability

The datasets used and/or analysed during the current study are available from the corresponding author upon reasonable request.
