# Supplementary material for: Molecular analysis of scats revealed diet and prey choice of grey wolves and Eurasian lynx in the contact zone between the Dinaric Mountains and the Alps
Source: Front Zool. 2024 Mar 19;21:9. doi: 10.1186/s12983-024-00530-6 (PMC10949697; doi:10.1186/s12983-024-00530-6)
Supplement: Supplementary file 2 — Additional file 2: Figure S1. Boxplots depicting the total number of predator and prey DNA reads for scat samples binned by the perceived degradation of the scat. Figure S2. NMDS plots of presence matrix Bray-Curtis dissimilarity of wolf and lynx scat samples, collected from different locations showing the prey taxa involved in driving distribution patterns. Figure S3. NMDS plots of RRA-based Bray-Curtis dissimilarity of wolf and lynx scat samples, collected from different locations showing the prey taxa involved in driving distribution patterns. Figure S4. Sampling locations in relation to density gradients of three ungulate (main prey) species in Slovenia: A Sus scrofa, B Capreolus capreolus, C Cervus elaphus. [file 12983_2024_530_MOESM2_ESM.docx]

**Supplemental Information for:**

**Molecular analysis of scats revealed diet and prey choice of grey wolves and Eurasian lynx in the contact zone between the Dinaric Mountains and the Alps**

Elena Buzan^1,2^, Hubert Potočnik^3^, Boštjan Pokorny^2,4^, Sandra Potušek^1^, Laura Iacolina^1,5^, Urška Gerič^1^, Felicita Urzi^1^, Ivan Kos^3^

**Table of Contents:**

| **Figure S1** | Page 2 |
| --- | --- |
| **Figure S2** | Page 3 |
| **Figure S3** | Page 4 |
| **Figure S4** | Page 5 |


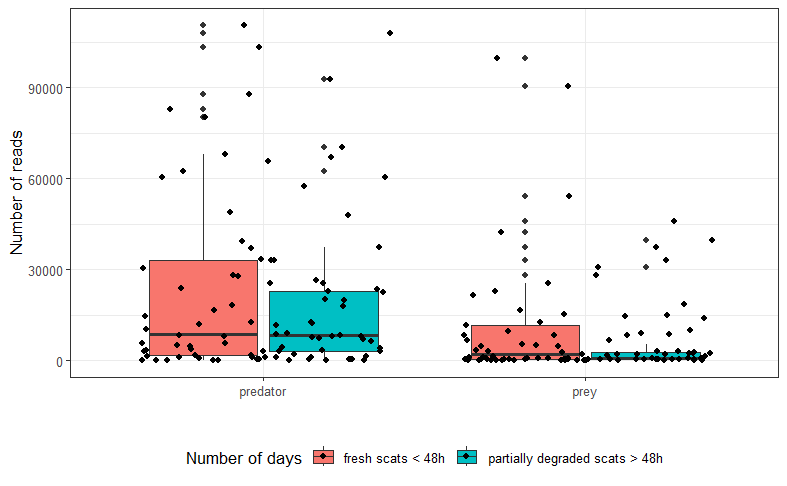


**Figure S1:** Boxplots depicting the total number of predator and prey DNA reads for scat samples binned by the perceived degradation of the scat.

**
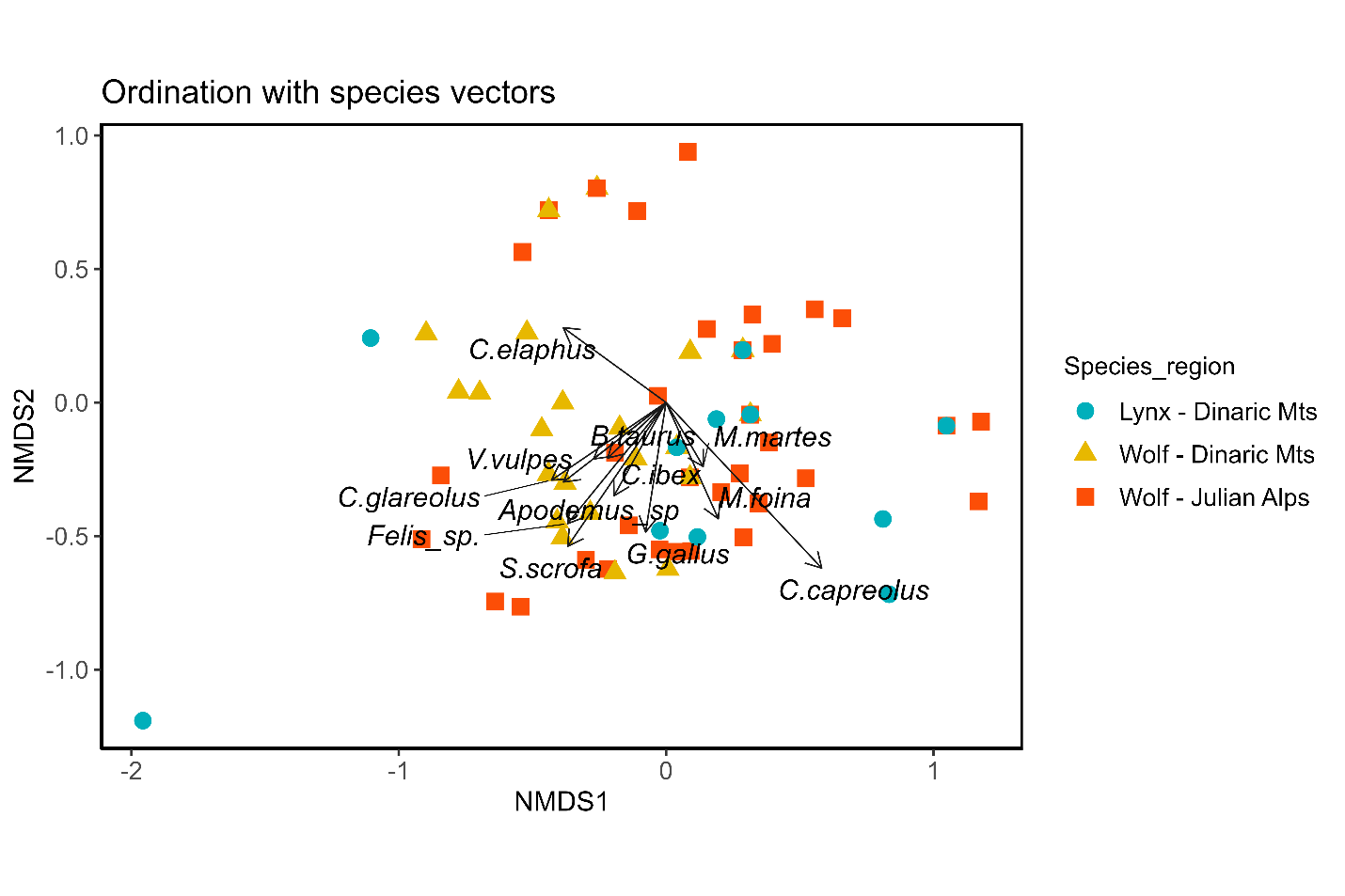
**

**Figure S2:** NMDS plots of presence matrix Bray-Curtis dissimilarity of wolf and lynx scat samples, collected from different locations (*p* = 0.001) showing the prey taxa involved in driving distribution patterns. The stress level of 0.163 is under the threshold value as suggested by Clark (1993) for an interpretable ordination.


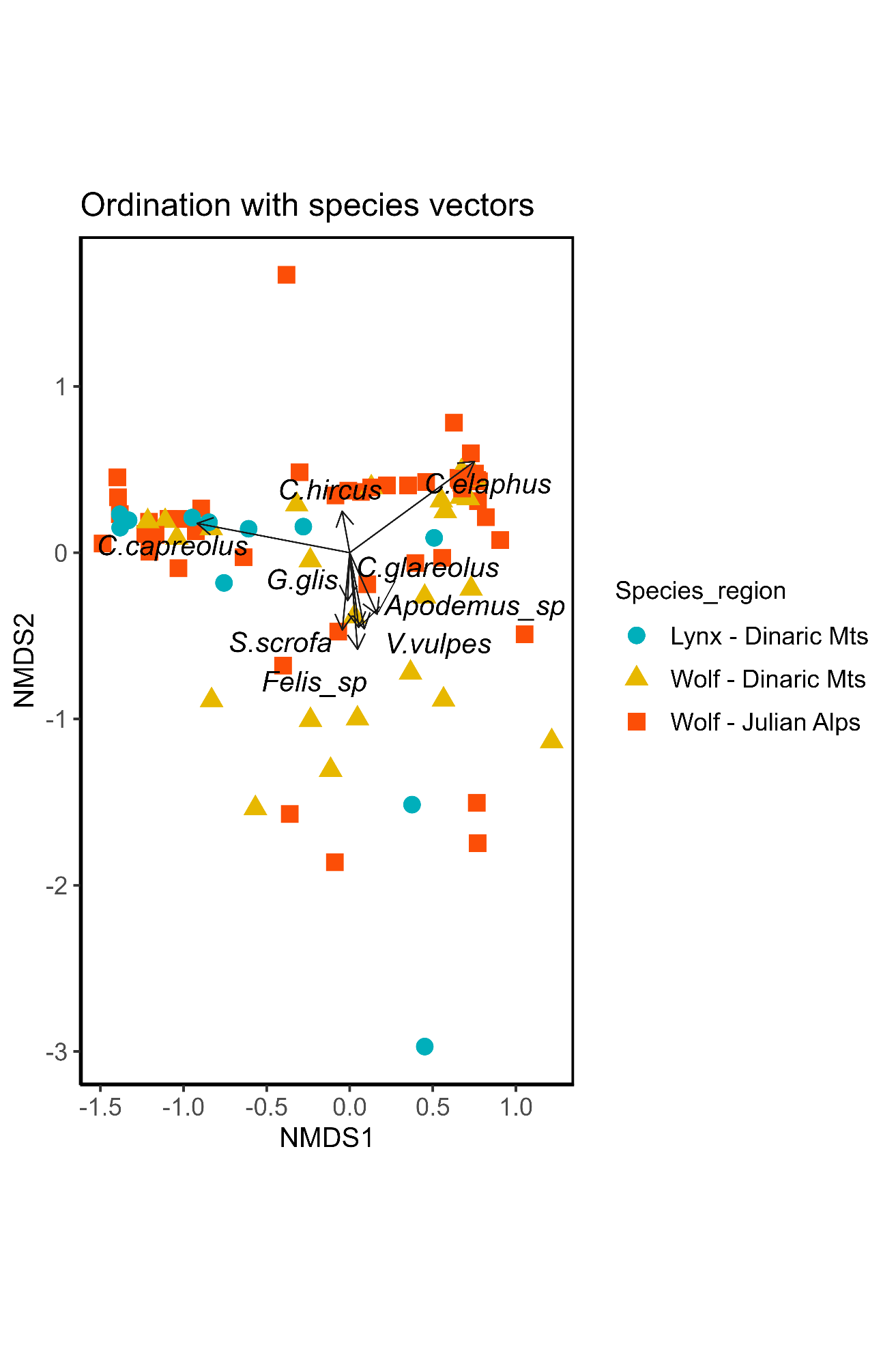


**Figure S3:** NMDS plots of RRA-based Bray-Curtis dissimilarity of wolf and lynx scat samples, collected from different locations (*p* = 0.001) showing the prey taxa involved in driving distribution patterns. The stress level of 0.149 is under the threshold value as suggested by Clark (1993) for an interpretable ordination.


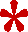

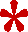

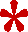

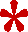


**A**


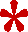
**
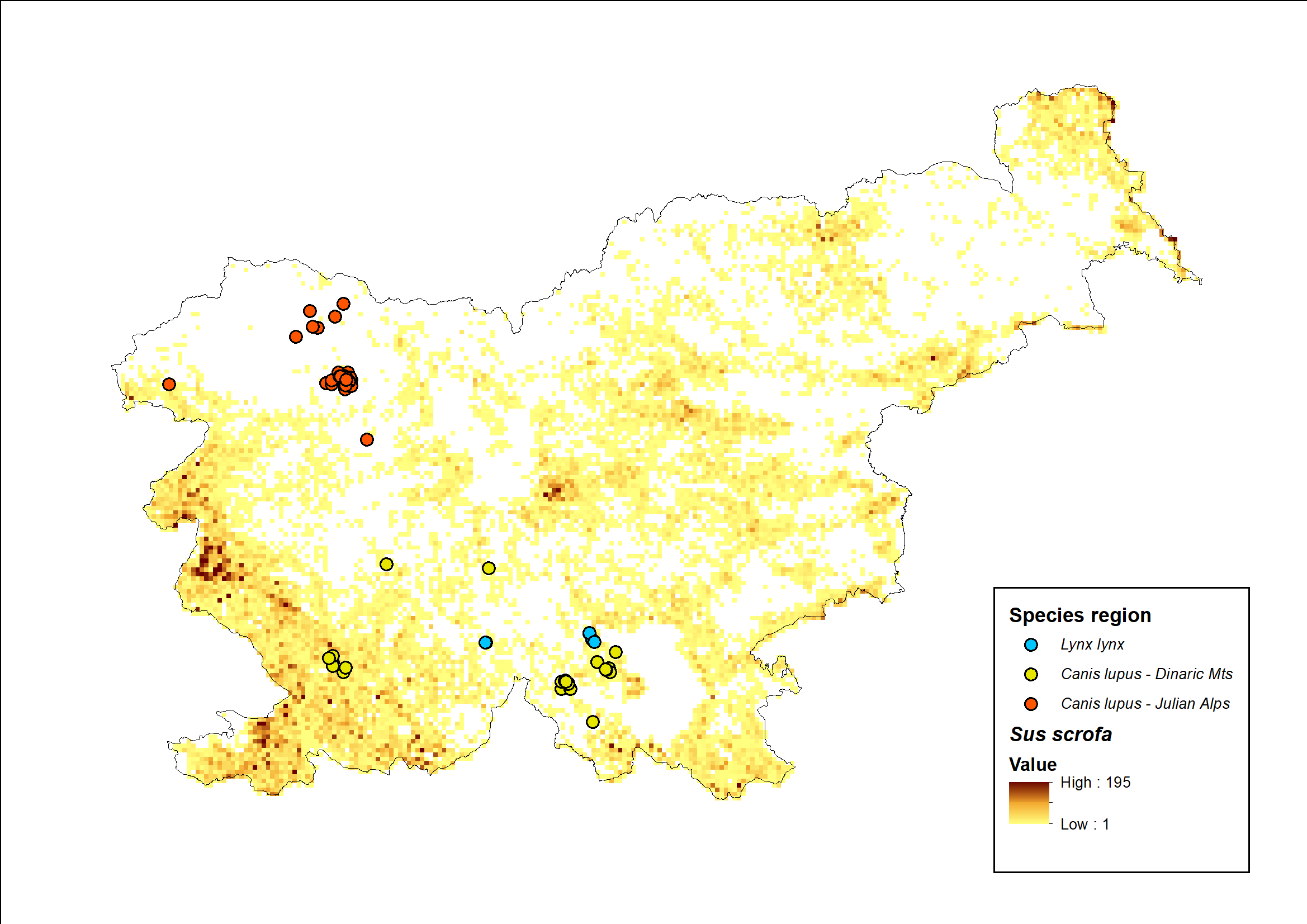
**

**B**


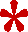
**
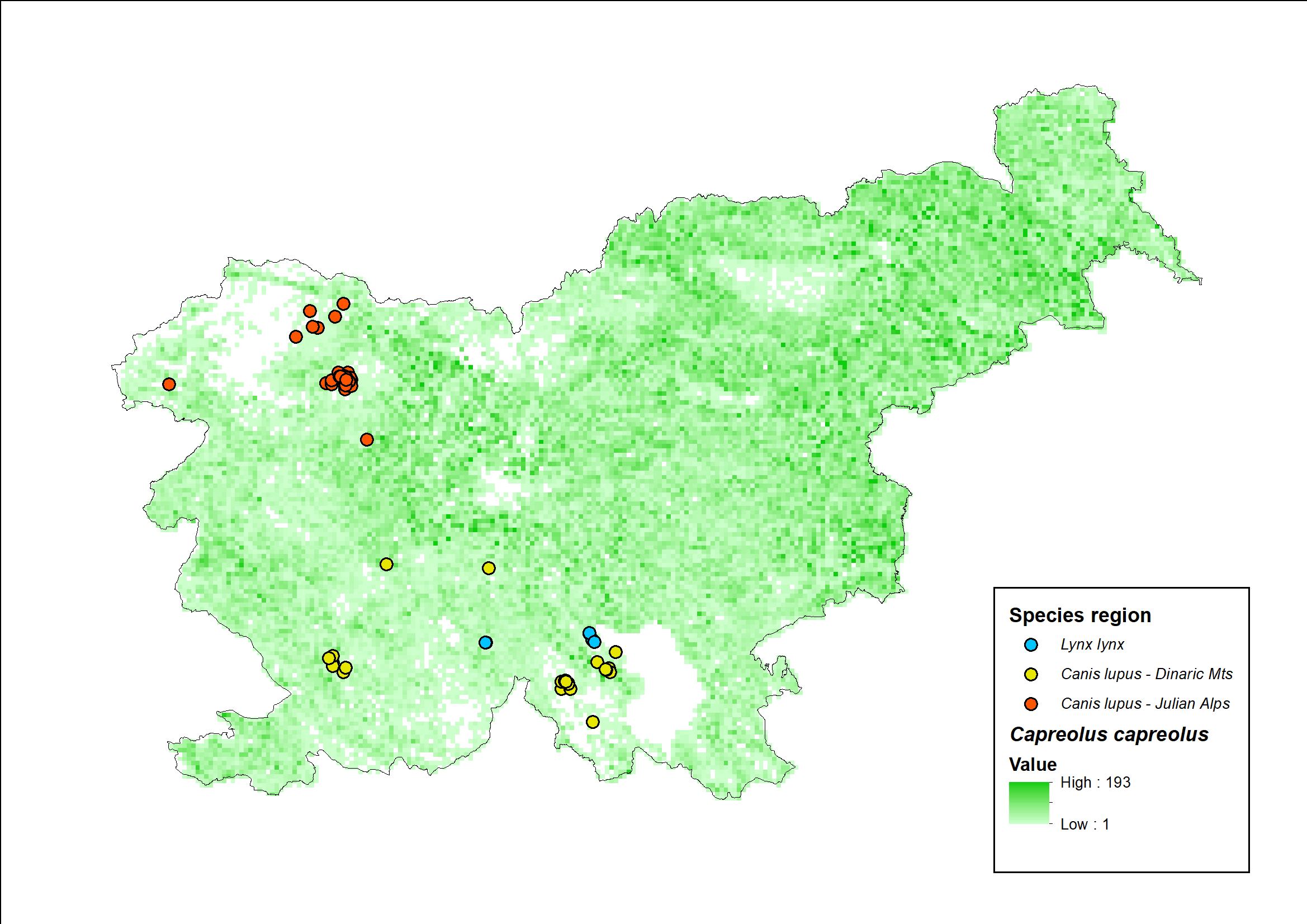
**

**C**


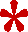
**
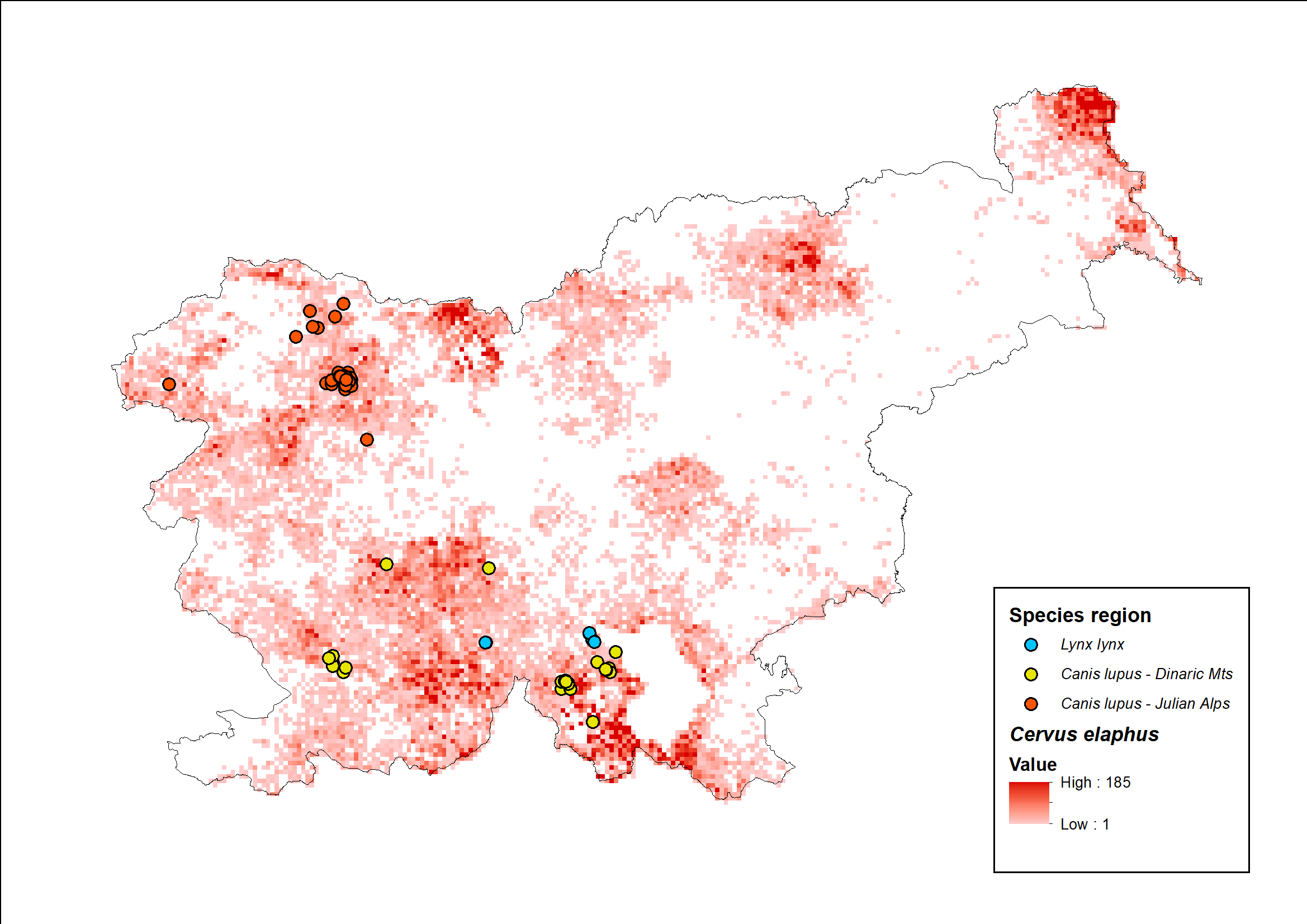
**

**Figure S4:** Sampling locations in relation to density gradients of three ungulate (main prey) species in Slovenia: A) *Sus scrofa*, B) *Capreolus capreolus*, C) *Cervus elaphus*. Density gradient for each species is presented based on comprehensive data on total registered mortality (harvest + losses, i.e. roadkill, diseases, predation etc.) of each species in high spatial resolution (1x1 km grid) in the period 2011–2020. Asterisks indicate two hunting grounds (41,400 ha in total) for which data is not available in adequate spatial resolution, therefore the area is shown in white, although population densities of all three ungulate species are there in spatial gradient similar as across neighbouring hunting grounds.
